# Downregulation of Bcl-2 sensitises interferon-resistant renal cancer cells to Fas

**DOI:** 10.1038/sj.bjc.6601895

**Published:** 2004-06-08

**Authors:** J D Kelly, J Dai, P Eschwege, J S Goldberg, B P Duggan, K E Williamson, N H Bander, D M Nanus

**Affiliations:** 1 Department of Oncology, Cambridge University, Box 193, Addenbrookes Hospital, Cambridge CB2 2QQ, UK; 2 Department of Urology, New York Presbyterian Hospital – Weill Medical College of Cornell University, 525 East 68 St, New York, NY 10021, USA; 3 Department of Urology, Bicetre Hospital, Paris-Sud University School of Medicine, France; 4 Uro-oncology Research Group, Cancer Research Centre, Institute of Pathology, Queens University Belfast, Grosvenor Rd, Belfast BT12 6BL, UK; 5 Division of Hematology and Oncology, Department of Medicine, New York Presbyterian Hospital – Weill Medical College of Cornell University, 525 East 68 St, New York, NY 10021, USA

**Keywords:** renal cell carcinoma, Fas, CD95, Bcl-2, interferon, apoptosis

## Abstract

Interferon *α* (IFN*α*) is used to treat patients with advanced renal cell carcinoma (RCC) despite limited clinical benefit. IFN*α* can induce Fas receptor-mediated apoptosis by direct activation of pro-caspase-8 followed by activation of caspase-3. Alternative, indirect activation of caspase-3 via mitochondrial release of cytochrome *c* can occur and may explain the rescue from Fas-activated cell death by the antiapoptotic members of the Bcl-2 family. In this study, we examined G3139, a novel antisense compound targeting Bcl-2, in combination with IFN*α*. Human RCC lines (SK-RC-44 and SK-RC-07) were treated with IFN*α*, G3139 or a combination of the two. Fas-mediated cytotoxicity was induced by anti-Fas mAb, CH11. An analysis of Bcl-2, Fas and the cleavage of PARP was performed. IFN*α* induced Fas and Bcl-2 in SK-RC-44 and SK-RC-07. IFN*α* sensitised SK-RC-44 to anti-Fas and induced PARP cleavage confirming that IFN*α* has a cytotoxic effect on RCC lines by induction of the Fas antigen. Cytotoxicity was not evident in SK-RC-07 cells treated with IFN*α*. G3139 induced a specific downregulation of Bcl-2 in SK-RC-07 cells, which were then sensitised to anti-Fas after treatment with IFN*α*. Taken together, these results suggest that Fas-dependent pathways as well as alternative pathways, which can be inhibited by Bcl-2, exist in renal cell carcinoma. G3139 in combination with IFN*α* is a potential therapy in patients with metastatic renal cell carcinoma.

Renal cell carcinoma (RCC) is the seventh leading cause of cancer accounting for 3% of all malignancies. Approximately one-third of patients with RCC have metastatic disease at presentation, and up to 50% relapse following nephrectomy (Stenzel and deKernion, 1989). The prognosis for patients with metastatic disease is unfavourable with a 3-year disease-free survival of less than 5%. Cytotoxic chemotherapy has demonstrated minimal activity for the treatment of RCC (Yagoda *et al*, 1995; Motzer *et al*, 1996). Reports of spontaneous regression of metastasis and prolonged treatment-free intervals for metastatic disease support the notion of an immunologically modulated disease. Immunological therapies such as interferon 2*α* (IFN*α*) and interlukin-2 are more effective than chemotherapy. Unfortunately, these therapies still only confer a limited survival advantage with complete response rates between 3 and 9% and partial response between 10 and 18% (reviewed in Figlin, 2000).

The mechanisms of resistance of RCCs to immunotherapy are not well defined. The Fas death receptor, which belongs to the tumour necrosis factor receptor family, is a key physiological regulator of cell death especially in immunologically mediated apoptosis (Nagata, 1999). In the kidney, Fas is constitutively expressed in the cells of the proximal tubules (progenitor cells for RCC) and also in RCC (Gerharz *et al*, 1999; Peduto *et al*, 1999). In cell culture experiments, the Fas pathway has been shown to be functional but requiring upregulation for activation of the cell death pathway (Nonomura *et al*, 1996; Gerharz *et al*, 1999; Lee JK *et al*, 2000). Cytokines including interferons have been shown to upregulate Fas in immortalised proximal tubular cells and RCC lines (Nonomura *et al*, 1996; Miyake *et al*, 1998; Sayers *et al*, 1998; Wu *et al*, 2000). Despite induced expression, cultured RCCs can remain resistant to apoptosis resulting from binding of agonistic anti-Fas antibodies to Fas receptor (Ramp *et al*, 2000).

Upon activation through binding to its natural ligand, the cytoplasmic region of the Fas receptor containing the death domain recruits and activates pro-caspase-8. Subsequently, depending on the cell type and/or stimulus, pro-caspase-8 can directly or indirectly activate caspase-3 to effect apoptosis (Scaffidi *et al*, 1998). In an indirect pathway, caspase-8 signalling is dependant on the mitochondria to transmit the apoptotic signal. Cytochrome *c* release from the mitochondria participates with apoptotic protease activating factor-1 in the activation of caspase-9, which in turn activates caspase-3 (Scaffidi *et al*, 1998; Zou *et al*, 1999). Recent evidence suggests that Fas receptor activation and apoptosis can be inhibited by Bcl-2 at the stage of cytochrome *c* release and caspase-9 activation and by binding and sequestering caspase-8 (Kawahara *et al*, 1998; Poulaki *et al*, 2001).

Bcl-2 is present in the distal collecting tubular cells of the normal kidney but upregulated in 20–50% of RCC, which develop from the proximal tubular cells. Bcl-2 expression has been shown to be present in a greater proportion of metastatic lesions when compared to the corresponding primary lesion (Huang *et al*, 1999; Lee CT *et al*, 2000; Zhang and Takenaka, 2000). In RCC cell lines, a reduction in Bcl-2 has been shown to be associated with increased sensitivity to anti-Fas (Hara *et al*, 2001). Based on these observations, we hypothesised that resistance to IFN*α* may be overcome by G3193, an antisense (AS) oligonucleotide targeting Bcl-2. We demonstrate that IFN*α* induces Fas and Bcl-2 in two RCC cell lines. Despite upregulation of Bcl-2, apoptosis evident by PARP cleavage was induced by anti-Fas in one cell line. In the resistant cell line, apoptosis could be induced by targeting Bcl-2 with G3139 and then stimulating with IFN*α*. The results suggest that G3139 in combination with IFN*α* may be a therapeutic strategy for patients with metastatic RCC.

## MATERIALS AND METHODS

### Cell culture

The human RCC lines, SK-RC-44 and SK-RC-07, were maintained in MEM/NEAA supplemented with 10% heat-inactivated fetal calf serum (FCS), 100 IU ml^−1^ penicillin, 100 M ml^−1^ streptomycin and 2 mM L-glutamine (Life Technologies, Grand Island, NY, USA). In most experiments, cells were plated in six-well tissue culture plates at a density of 5 × 10^5^ and were allowed to adhere overnight. For cell viability studies, cells were plated at a density of 4 × 10^3^ cells per well in 96-well plates (Becton Dickinson, Franklin, NJ, USA). Cells were incubated in either medium or in medium containing the indicated concentrations of recombinant human interferon 2*α*b (IFN*α*) (Schering, Keniworth, NJ, USA) for the specified times.

### Fas-induced cell death

Fas-mediated cytotoxicity was induced by addition of the agonistic crosslinking anti-Fas mAb, CH11 (Immunotech, Marseille, France). For dose–response experiments, cells were incubated with CH11 at the indicated concentrations; otherwise, CH11 was used at a concentration of 50 ng ml^−1^. An antagonistic anti-Fas antibody, ZB4 (Immunotech, Marseille, France), which inhibits apoptosis induced by CH11, was added where specified at a concentration of 1 *μ*g ml^−1^ 1 h before the addition of the CH11 antibody. Caspase activity was determined by the cleavage of poly(ADP–ribose) polymerase (PARP) detected by Western blotting.

### G3139

G3139 (kindly provided by Genta Inc.), an 18-mer phosphothioate AS targeted at the translation initiation site of the Bcl-2 mRNA, was used. The G3139, sequence 5′-tctcccagcgtgcgccat-3′ as well as reverse sense (RS), sequence 5′-taccgcgtgcgaccctct-3′ and missense (MS), sequence 5′-tctcccagcatgtgccat-3′ controls were obtained from Genta (Boston, MA, USA). All of the oligonucleotides were shipped as a lyophilised powder after purification by grade 1 HPLC. When reconstituted, the stock solution was stored at −20°C. Oligonucleotides were combined with a transfection agent before use. The agent used was a liposome formulation of cationic lipids Eu.Fectin™ GC-030/DOPE in a 2 : 1 ratio (Promega, San Luis Obispo, CA, USA). A lipid solution was made using serum-free medium (Opti-MEM, Life Technologies, Grand Island, NY, USA) so that its concentration equalled 2 × the concentration of the oligonucleotide. The Eu.Fectin-Opti-MEM solution was allowed to incubate at room temperature for 30–45 min so that optimal formation of liposomes was achieved. It was then combined with the oligonucleotides for cell transfections.

### Western blotting

Cells were washed in phosphate-buffered saline (PBS), scraped and then lysed at 4°C with RIPA buffer containing 1% Nonidet P40, 1% deoxycholate, 0.1% SDS supplemented with 8 *μ*g ml^−1^ aprotinin, 2 *μ*g ml^−1^ leupeptin and 170 *μ*g ml^−1^ phenylmethylsulphonyl fluoride (PMSF) for 30 min. After centrifugation, the protein concentration of the supernatant was determined by colorimetric analysis using the Bio-Rad protein assay system (Bio-Rad Laboratories, Richmond, CA, USA). Equal quantities of lysates (25 *μ*g) were separated on either 12% (Bcl-2, Bcl-X_L_) or 7.5% (PARP) SDS–PAGE under reducing conditions and transferred onto enhanced chemiluminescence (ECL) membranes (Amersham, Arlington Heights, IL, USA). Membranes were then blocked with 5% milk before incubation with specific antibodies: M0887 Bcl-2 mouse anti-human (Dako), AM05 Bcl-xl mouse anti-human (Calbiochem, San Diego, CA, USA), 65196E PARP mouse anti-human (Pharmingen, San Diego, CA, USA) and MAB1501 *β*-actin (Chemicon, Temecula, CA, USA). Bound antibodies were detected with an anti-mouse/horseradish peroxidase conjugate (Amersham). An ECL system (Amersham) was used for detection.

### Flow cytometry for Fas

Cells were harvested by scraping, washed once in 2% FCS–MEM NEAA (staining media) at 4°C and then resuspended in staining media containing 1 mg ml^−1^ bovine serum albumin for 30 min. The cells were pelleted and then resuspended in 2% FCS–PBS containing ZB4 mouse monoclonal anti-Fas (1 : 50 dilution) or isotype control, IgG1 clone 679.1Mc7 (Immunotech, Marseille, France) for 30 min at 4°C. The cells were washed once in staining media and then incubated with FITC-labelled anti-murine IgG (0810 Immunotech, Marseille, France) in staining media for 30 min at 4°C. The cells were washed twice in 2% FCS–PBS and analysed immediately on the flow cytometer. At least 10 000 cells were analysed in each sample and experiments were performed in triplicate.

### MTT assay

RCC cells were plated at a density of 4 × 10^3^ cells per well in 96-well plates (Becton Dickinson, Franklin, NJ, USA). After 24 h, the culture medium was replaced with medium containing IFN*α* at the specified concentrations or with culture medium alone. Viability was determined by use of a colorimetric assay, based on the reduction of the tetrazolium salt MTT (3-[4,5-dimethylthiazol-2-yl]-2,5-diphenyltetrazolium bromide), by mitochondrial dehydrogenases, in viable cells (Morgan, 1998). After incubations for 48 or 96 h, medium was replaced with 200 *μ*l MTT reagent (Sigma, St Louis, MO, USA) (0.5 mg ml^−1^) and allowed to react for 4 h at 37°C. The solubilising agent dimethyl sulphoxide (DMSO, Fisher Scientific, Fairlawn, NJ, USA), 100 *μ*l well^−1^, was added and the substrate cleavage was determined at 560 nm on a SPECTRAmax 340 microplate reader and analysed using SOFTmax PRO software (Molecular Devices, Sunnnyvale, CA, USA).

## RESULTS

### IFN*α* upregulated Bcl-2 and Fas expression

**Figure 1:**
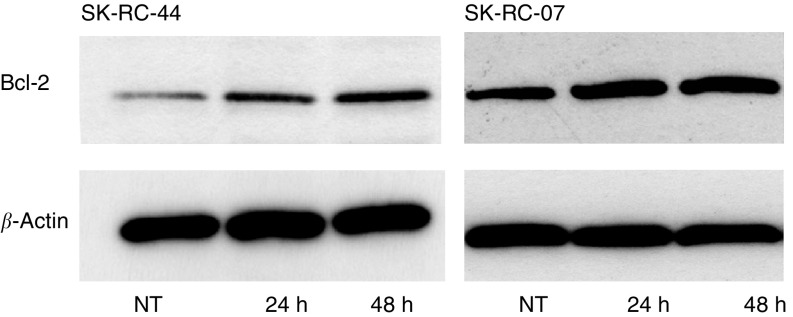
Western blot analysis of Bcl-2 protein in cells treated with IFN*α*. SK-RC-44 and SK-RC-07 were incubated with 1000 U ml^−1^ IFN*α* for 24 and 48 h. NT, no treatment control. Protein samples (25 *μ*g lane^−1^) were probed with an anti-Bcl-2 antibody. In both cell lines, IFN*α* induced Bcl-2 expression within 24 h and levels were sustained at 48 h. Equal protein loading is shown by reproducing the identical blot with anti-*β*-actin antibody.

**Figure 2:**
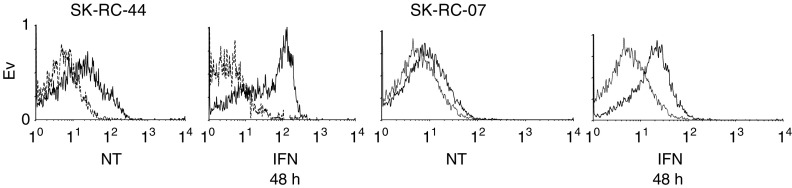
Flow cytometry analysis of Fas in cells treated with IFN*α*. SK-RC-44 and SK-RC-07 cell lines were incubated with IFN*α* (1000 U ml^−1^) for the indicated times. Cell surface staining of Fas was determined in no treatment (NT) controls and 48 h after IFN*α* treatment. Representative overlay histograms; dotted line IgG1 isotype control antibodies; solid line, Fas antibodies. Fas was constitutively expressed in SK-RC-44 and SK-RC-07 cells. There was a significant rise in the percentage of Fas-positive SK-RC-44 cells, 34.8±5.4 *vs* 68.5±6.4 (before and after IFN*α* treatment, respectively) (*P*<0.003). In SK-RC-07, Fas increased from 21.8±4.5 to 36.1±8.1 following incubation with IFN*α* (*P*=0.03).

Bcl-2 protein was constitutively expressed by both SK-RC-44 and SK-RC-07 cells as detected by Western blotting (Figure 1

). Expression of Bcl-2 increased within 24 h of IFN*α* treatment and this was sustained at 48 h (Figure 1). Fas antigen was constitutively expressed and incubation with IFN*α* for 48 h increased Fas expression in SK-RC-44 and SK-RC-07 (Figure 2

).

### Effects of anti-Fas mAb and IFN on cell viability

**Figure 3:**
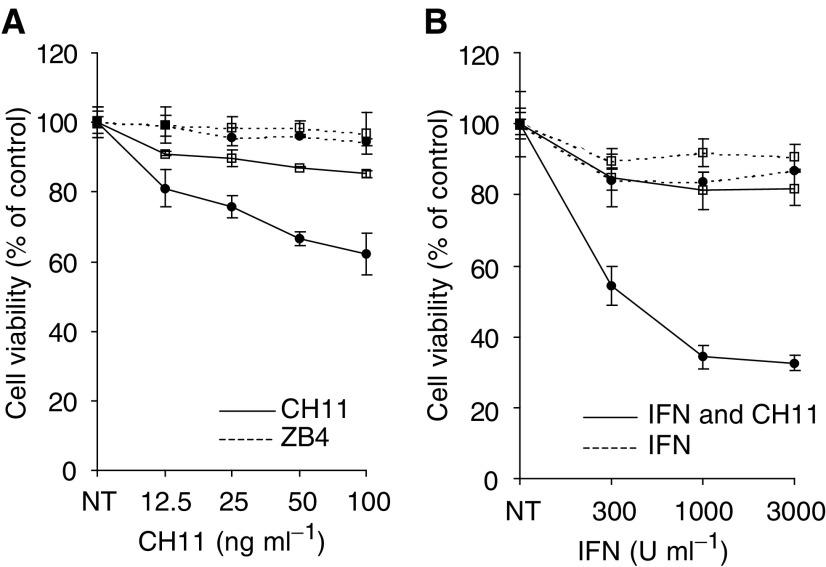
Analysis of anti-Fas antibody and IFN*α* on the viability of SK-RC-44 (•) and SK-RC-07 (□) cells by MTT assay. (**A**) Anti-Fas antibody CH11 at various concentrations was added to cells that were cultured in 96-well plates (solid line). The antagonistic anti-Fas mAb ZB4 (1 *μ*g ml^−1^) was added to the culture medium of cells 1 h before CH11 (dashed line). Cell viability was determined after 24 h. (**B**) Cells were cultured with IFN*α* at the indicated concentrations for 72 h (dashed line), or with IFN*α* for 72 h and CH11 (50 ng ml^−1^), which was added to each well after 48 h (solid line). Results are expressed as percentage of nontreated control cells (NT). CH11 was cytotoxic for SK-RC-44, which exhibited a dose response to the antibody. The loss of cell viability of SK-RC-44 could be blocked by ZB4. The effect of CH11 on SK-RC-44 cells was markedly enhanced by pretreatment with IFN*α*. In comparison, the response of SK-RC-07 cells to CH11 was limited and not enhanced by pretreatment with IFN*α*.

Fas induction by IFN*α* prompted examination of the effect of the anti-Fas antibody (CH11) on cell viability of SK-RC-44 and SK-RC-07 cells. In a dose–response study conducted over 24 h, SK-RC-44 cells exhibited a dose-dependent loss of viability to CH11 over 24 h, which could be blocked by preincubation with the antagonistic anti-Fas monoclonal antibody ZB4. In contrast, SK-RC-07 cells did not exhibit a significant response to CH11 (Figure 3A

).

As shown in Figure 3B, treatment with IFN*α* alone for 72 h had little effect on either cell line. However, pretreatment of SK-RC-44 with IFN*α* for 48 h followed by CH11 for 24 h markedly enhanced the anti-Fas-mediated cytotoxicity. This effect was not apparent in SK-RC-07 in which there was only a modest cytotoxic effect of CH11 (Figure 3B).

### SK-RC-44 cells but not SK-RC-07 can activate Fas-dependent cleavage of PARP

**Figure 4:**
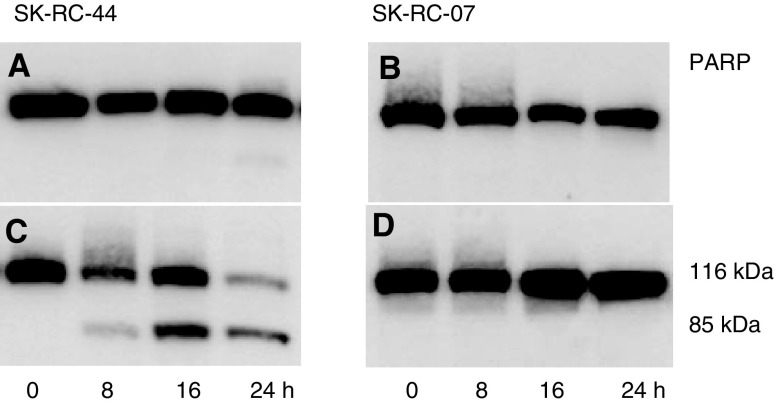
Analysis of PARP cleavage by Western blot. SK-RC-44 and SK-RC-07 cells were incubated with CH11 (50 ng ml^−1^) (**A**, **B**), or pretreated with IFN*α* for 48 h followed by CH11 (50 ng ml^−1^) for 8, 16 or 24 h (**C**, **D**). PARP cleavage was determined at the indicated time points by Western blot as described in Materials and methods. IFN*α* did not induce PARP cleavage in either cell line. CH11 induced a weak PARP cleavage in SK-RC-44 cells but only after 24 h (**A**). In SK-RC-44 cells pretreated with IFN*α*, cleavage of PARP was obvious and occurred within 8 h of CH11 being added to the medium (**C**). In the SK-RC-07 cells, neither treatment with CH11 (**B**) nor with IFN*α* and CH11 (**D**) induced PARP cleavage.

When induction of Fas by IFN*α* was followed by Fas ligation with CH11, there was a significant cytotoxic effect in SK-RC-44 cells compared to only a modest cytotoxic effect in SK-RC-07. To determine whether cell death was through apoptosis, PARP cleavage was examined. PARP-1 is the target of caspase-3, which cleaves PARP-1 within a DEVD site in the DNA-binding domain, thus splitting the nuclear localisation sequence into two detectable fragments. The activation of caspase-3 proteases in response to Fas/FasL interaction was determined by the cleavage of PARP into 89 and 24 kDa fragments (Figure 4

) (Lazebnik *et al*, 1994). IFN*α* alone did not cleave PARP in either cell line. When cells were incubated with only CH11, the appearance of a very weak 89 kDa band indicated that anti-Fas induced PARP cleavage in SK-RC-44 cells after 24 h. Pretreatment of SK-RC-44 with IFN*α* for 48 h followed by CH11 did induce PARP cleavage, which was evident after 8 h and occurred throughout the 24 h. In SK-RC-07 cells, CH11 alone, or following pretreatment of cells with IFN*α,* did not induce PARP cleavage.

These data show that failure of the Fas cell death pathway in SK-RC-07 occurred despite demonstrating that transcription of Fas mRNA and induction of the Fas antigen. We postulated that the upregulation of Bcl-2 may inhibit the cytotoxic effect of IFN*α* and also prevent cells from responding to the apoptotic stimulus of CH11.

### Downregulation of Bcl-2 with AS G3139

**Figure 5:**
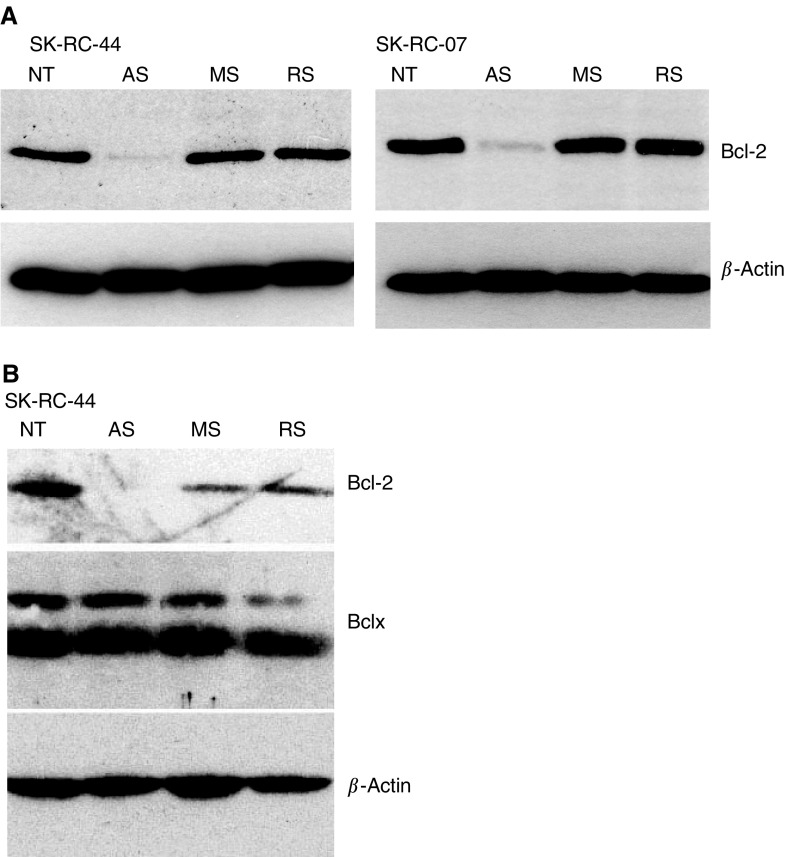
Western blot analysis of Bcl-2 following treatment with oligonucleotides. (**A**) SK-RC-44 and SK-RC-07 cells were plated in six-well plates for 24 h (5 × 10^5^ cells well^−1^). Cells were incubated with Eu.Fectin/oligonucleotide G3139 AS, MS or RS oligonucleotides at a concentration of 300 nM for 4 h. NT, no treatment controls. After 24 h, Bcl-2 expression was determined by Western blot. Bcl-2 was downregulated by G3139 AS in SK-RC-44 and SK-RC-07. At the concentration of 300 nM, MS or RS did not cause a significant reduction in Bcl-2 expression in either cell line. (**B**) Following detection of Bcl-2, a membrane was stripped and re-probed for Bclx protein. Bcl-2 is downregulated by G3139 AS but Bclx expression is unaffected.

To determine whether downregulation of Bcl-2 sensitised cells to the IFN*α*/Fas stimulus, cells were transfected with a Bcl-2 AS oligonucleotide, G3139. Transmission of the apoptotic signal following Fas ligation may follow either a direct caspase-8 activation of caspase-3 pathway or an indirect pathway with a mitochondrial intermediate step. Expression of Bcl-2 protein can inhibit the indirect pathway. Cell transfection rates >80% were achieved using a single lipid (GC-030/DOPE). A dose–response study in SK-RC-07 cells demonstrated effective downregulation of Bcl-2 by G3139 with little effect of MS or RS controls and less than 10% loss of cell viability at oligonucleotide concentrations below 300 nM and an Eu.Fectin:oligo ratio of 2 : 1 (data not shown). Figure 5A

demonstrates the downregulation of Bcl-2 in SK-RC-44 and SK-RC-07 cells 24 h after incubation with G3139. Expression of the Bclx protein, which shares significant sequence homology with Bcl-2, was not affected, demonstrating target specificity of the G3139 oligonucleotide (Figure 5B).

### Downregulation of Bcl-2 sensitises IFN*α*-treated cells to anti-Fas

**Figure 6:**
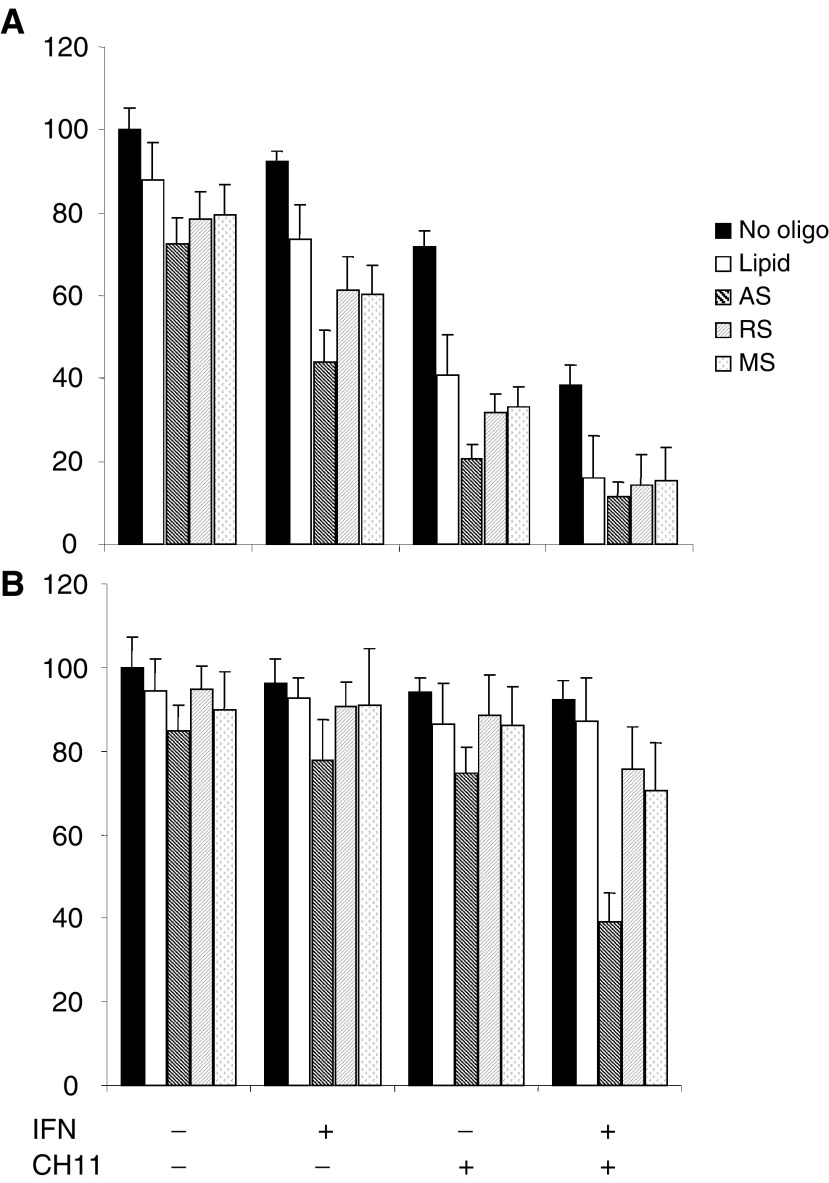
Cell viability following anti-Fas in oligonucleotide- and IFN*α*-treated cells. SK-RC-44 (**A**) and SK-RC-07 (**B**) cells were cultured in 96-well plates and incubated with G3139 AS or controls as described in Materials and methods. After 24 h, cells were incubated with IFN*α* (1000 U ml^−1^) for 72 h, or with IFN*α* for 48 h followed by CH11 (50 ng ml^−1^) for 24 h. Cell viability was assessed by MTT. Results are expressed as a percentage of untreated controls. Single-agent treatment with G3139 reduced the viability of both SK-RC-44 and SK-RC-07 cells but was not significant when compared to the effect of MS and RS. In SK-RC-44, G3139 followed by CH11 reduced the viability of cells to 20.7±3.2 and was significantly greater than the effect of MS or RS (*P*<0.001). In cells incubated with G3139 and then treated with IFN*α* and CH11, viability was reduced to 11.2±3.4 but an effect of MS and RS controls was also evident. The effect of G3139 in combination with IFN*α* or CH11 did enhance the effect of both agents in SK-RC-07 cells, but the effect was limited. There was however a synergistic effect when SK-RC-07 cells were incubated with G3139 and then treated with IFN*α* followed by CH11. Cell viability was reduced to 38.7±7.2 by this combination and was significantly greater than in controls.

In 96-well plates, SK-RC-44 and SK-RC-07 cells were incubated with G3139, MS, RS or empty lipid controls. After 24 h, cells were treated with IFN*α* for a further 48 h followed by CH11 as previously described. Single-agent treatment with G3139 reduced the viability of both SK-RC-44 and SK-RC-07 cells, and the reduction was not significant when compared to the effect of MS and RS or empty lipid (Figure 6

). Incubation with G3139 followed by IFN*α* reduced the viability of SK-RC-44 cells (43.9±7.9) (Figure 6A). An effect of MS, RS or empty lipid controls was also seen (cell viability 60.3±6.9, 61.6±7.8 and 73.6±8.5, respectively). The effect of G3139 and IFN*α* was significantly greater than controls in SK-RC-44 cells (*P*<0.01). G3139 followed by CH11 reduced the viability of SK-RC-44 cells to 20.7±3.2, which was significantly greater than MS, RS or empty lipid (*P*<0.001). In cells incubated with G3139 and then treated with IFN*α* and CH11, viability was reduced to 11.2±3.4 but an effect of MS and RS controls was also evident.

The effect of G3139 on the IFN*α*/Fas-resistant SK-RC-07 cells is shown in Figure 6B. G3139 alone did not cause a significant loss of viability compared to MS or RS controls. G3139 in combination with IFN*α* or CH11 did enhance the effect of both agents and was significantly greater than controls; however, the effect was not significant (G3139 and IFN*α* 77.8±9.6 and G3139 and CH11 74.6±6.1). There was a synergistic effect when SK-RC-07 cells were incubated with G3139 and then treated with IFN*α* followed by CH11. The combination reduced cell viability to 38.7±7.2 (MS 75.4±9.7 and RS 70.6±11.2) (*P*<0.01).

### Fas-resistant SK-RC-07 cells cleave PARP upon downregulation of Bcl-2

**Figure 7:**
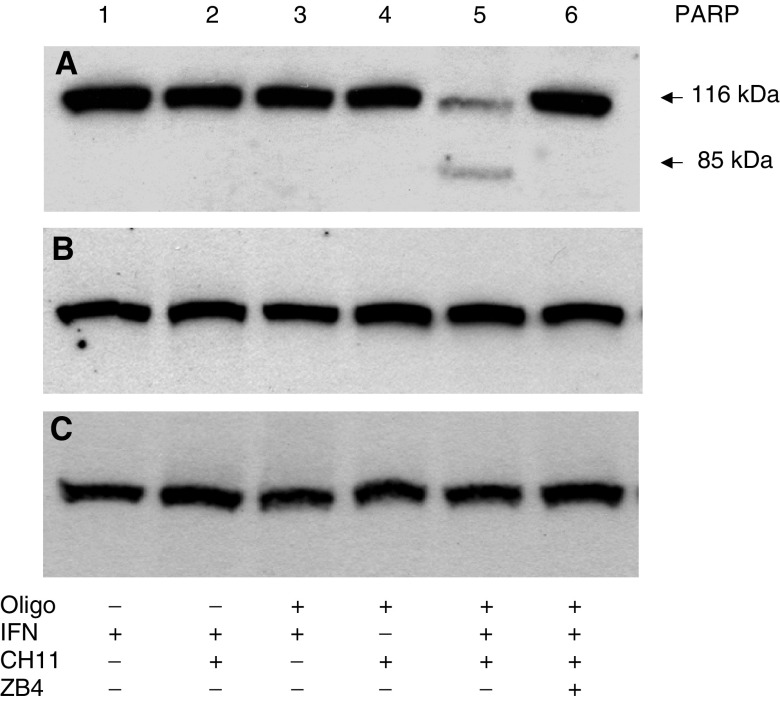
Analysis of PARP cleavage by Western blot. SK-RC-07 cells were incubated with G3139 AS (**A**), MS (**B**) or RS (**C**) oligonucleotides. Cells were treated with IFN*α* (1000 U ml^−1^) for 48 h and then by CH11 (50 ng ml^−1^) for 24 h as indicated. ZB4 was added to cells 1 h before treatment with CH11 (lane 6). PARP cleavage was determined by Western blot as described in Materials and methods. SK-RC-07 cells treated with G3139 were sensitive to the Fas antibody CH11 but only when pretreated with IFN*α* (lane 5). Neither treatment with mismatch or RS sensitised cells to Fas-induced PARP cleavage. PARP cleavage could be inhibited by the addition of ZB4 before treatment with CH11 (lane 6).

To determine if anti-Fas would cleave PARP in Bcl-2 downregulated cells, SK-RC-07 cells were incubated with AS or controls and then IFN*α* and CH11 (Figure 7

). PARP was only cleaved by CH11 in cells pretreated with G3139 followed by IFN*α*. G3139/IFN*α* and G3139/CH11 had no effect and in addition, PARP cleavage did not occur in MS or RS controls. Furthermore, cleavage of PARP by CH11 in G3139/IFN*α*-treated cells was inhibited by the addition of ZB4. These results suggest that downregulation of Bcl-2 permits apoptosis to occur following Fas/FasL interaction in IFN*α*-treated cells.

## DISCUSSION

Recombinant IFN*α* has created the first window of opportunity for the treatment of metastatic RCC. However, complete response rates to IFN*α* and/or other immunological agents remain limited. To date, it is unclear whether IFN*α* effects are mediated by the known direct antiproliferative activity in RCC lines or by a direct cytotoxic effect (Figlin, 1999).

We have shown that RCC lines constitutively express Fas and that IFN*α* upregulates Fas as well as the apoptosis-inhibiting Bcl-2 protein. Mechanistically, the observations can be explained by studies showing that IFNs can activate nuclear factor *κ*B through an RNA-dependent protein kinase resulting in transcription of *bcl-2* genes as well as Fas (Lee HH *et al*, 1999; Tan and Katze, 1999; Harwood *et al*, 2000).

IFN*α* did not have a direct cytotoxic effect on either of our cell lines; however, cleavage of PARP was evident in IFN*α*-treated SKRC-44 cells when incubated with anti-Fas antibody CH11. Our results are in keeping with reports that upregulation of Fas is a prerequisite for sensitisation of cells to anti-Fas-mediated apoptosis (Gerharz *et al*, 1999; Lee JK *et al*, 2000). PARP is one substrate for pro-caspase-8, suggesting that the effect of IFN*α* was to induce Fas and that the Fas pathway was subsequently activated and intact. PARP cleavage was not evident in SKRC-07 cells despite a significant induction of Fas by IFN*α*. Fas expression was higher in SKRC-44 cells, which could explain the differential sensitivity compared to SKRC-04. That SKRC-07 cells were not responsive to anti-Fas could be a consequence of the lower level of Fas expression even after induction by IFN*α*. We have shown that IFN*α* induces Bcl-2 in SKRC-07 cells, which led us to postulate that Bcl-2 rescue of cells could prevent Fas-induced apoptosis. It has been recently shown that different cell lines can respond differently to cell death induced by anti-Fas. One class of cells activates caspase-8 and caspase-3 efficiently and independent of mitochondrial signals. For this reason, cells are not rescued by Bcl-2 expression. Other cells do not strongly activate caspase-8 and must use a signalling pathway that is dependent on mitochondrial death signals and are thus inhibited by Bcl-2 expression (Scaffidi *et al*, 1998; Johnson and Boise, 1999). This concept can explain the reports that IFN*α* can inhibit apoptosis in some cells (Su and David, 1999) and, alternatively, induce apoptosis in other cells (Lee RK *et al*, 1999). To determine whether inhibition of Bcl-2 expression would sensitise the Fas-resistant cells to anti-Fas, we used an AS oligonucleotide, G3139. Bcl-2 was effectively downregulated by G3139 but not mismatch and RS controls and target specificity was demonstrated by the preservation of Bclx expression. Downregulation of Bcl-2 sensitised SKRC-07 cells to anti-Fas, which was evident following induction of Fas with IFN*α*. In these cells, PARP cleavage was inhibited by the antagonistic antibody ZB4, suggesting that ultimately the cell death stimulus is initiated through a Fas-dependent pathway. In the Fas-sensitive SKRC-44 cell line, downregulation of Bcl-2 is an additional but nonspecific cytotoxic event. AS reduces the cell viability, but this effect is also seen in RS and MS controls. An explanation for this effect may be that the transfection of oligo/lipid simply adds insult to injury in an already dying cell. Interestingly, in the Fas-resistant SKRC-07 cells, the downregulation of Bcl-2 did not induce a loss of cell viability to the extent seen in the sensitive line treated with IFN*α* and anti-Fas. It is possible that preservation of other apoptosis-inhibiting Bcl-2 family proteins maintains the survival signal.

The Fas-mediated apoptosis pathway is present and inducible in proximal tubular cells of the normal kidney (Jo *et al*, 2002). This study has confirmed that IFN*α* has a cytotoxic effect on RCC lines by induction of the Fas antigen. The *in vitro* observation might explain the complete response rates seen in some patients following IFN*α* therapy. G3139 effectively reduced Bcl-2 expression and enabled Fas-induced apoptosis in a resistant cell line with low levels of Fas expression. Cell death was Fas dependent and supports the notion that some cells can activate alternative Fas pathways, which can be inhibited by Bcl-2. These pathways may be present to a greater or lesser extent within different cell lines of RCC. Recently, Bcl-2 has been shown to be upregulated in a proportion of renal cell metastatic lesions as compared to the primary lesions (Lee CT *et al*, 2000). This finding could be a factor responsible for the resistance of RCC to IFN*α* therapy. The demonstration that sensitivity to Fas apoptosis can be enhanced by downregulation of Bcl-2 suggests that the effectiveness of IFN*α* can be improved *in vivo*.
